# Impact of providing patients access to electronic health records on quality and safety of care: a systematic review and meta-analysis

**DOI:** 10.1136/bmjqs-2019-010581

**Published:** 2020-06-12

**Authors:** Ana Luisa Neves, Lisa Freise, Liliana Laranjo, Alexander W Carter, Ara Darzi, Erik Mayer

**Affiliations:** 1 Patient Safety Translational Research Centre, Institute of Global Health Innovation, Imperial College London, London, UK; 2 Center for Health Technology and Services Research / Department of Community Medicine, Health Information and Decision (CINTESIS/MEDCIDS), Faculty of Medicine, University of Porto, Porto, Portugal; 3 Westmead Applied Research Centre, Faculty of Medicine and Health, University of Sydney, Sydney, New South Wales, Australia; 4 Centre for Health Informatics, Australian Institute of Health Innovation, Sydney, New South Wales, Australia; 5 Department of Health Policy, London School of Economics & Political Science, London, UK

**Keywords:** patient safety, health policy, patient-centred care, information technology

## Abstract

**Objective:**

To evaluate the impact of sharing electronic health records (EHRs) with patients and map it across six domains of quality of care (ie, patient-centredness, effectiveness, efficiency, timeliness, equity and safety).

**Design:**

Systematic review and meta-analysis.

**Data sources:**

CINAHL, Cochrane, Embase, HMIC, Medline/PubMed and PsycINFO, from 1997 to 2017.

**Eligibility criteria:**

Randomised trials focusing on adult subjects, testing an intervention consisting of sharing EHRs with patients, and with an outcome in one of the six domains of quality of care.

**Data analysis:**

The Preferred Reporting Items for Systematic Reviews and Meta-Analyses guidelines were followed. Title and abstract screening were performed by two pairs of investigators and assessed using the Cochrane Risk of Bias Tool. For each domain, a narrative synthesis of the results was performed, and significant differences in results between low risk and high/unclear risk of bias studies were tested (t-test, p<0.05). Continuous outcomes evaluated in four studies or more (glycated haemoglobin (HbA1c), systolic blood pressure (SBP) and diastolic blood pressure (DBP)) were pooled as weighted mean difference (WMD) using random effects meta-analysis. Sensitivity analyses were performed for low risk of bias studies, and long-term interventions only (lasting more than 12 months).

**Results:**

Twenty studies were included (17 387 participants). The domain most frequently assessed was effectiveness (n=14), and the least were timeliness and equity (n=0). Inconsistent results were found for patient-centredness outcomes (ie, satisfaction, activation, self-efficacy, empowerment or health literacy), with 54.5% of the studies (n=6) demonstrating a beneficial effect. Meta-analyses showed a beneficial effect in effectiveness by reducing absolute values of HbA1c (unit: %; WMD=−0.316; 95% CI −0.540 to −0.093, p=0.005, I^2^=0%), which remained significant in the sensitivity analyses for low risk of bias studies (WMD= −0.405; 95% CI −0.711 to −0.099), and long-term interventions only (WMD=−0.272; 95% CI −0.482 to −0.062). A significant reduction of absolute values of SBP (unit: mm Hg) was found but lost in sensitivity analysis for studies with low risk of bias (WMD= −1.375; 95% CI −2.791 to 0.041). No significant effect was found for DBP (unit: mm Hg; WMD=−0.918; 95% CI −2.078 to 0.242, p=0.121, I^2^=0%). Concerning efficiency, most studies (80%, n=4) found either a reduction of healthcare usage or no change. A beneficial effect was observed in a range of safety outcomes (ie, general adherence, medication safety), but not in medication adherence. The proportion of studies reporting a beneficial effect did not differ between low risk and high/unclear risk studies, for the domains evaluated.

**Discussion:**

Our analysis supports that sharing EHRs with patients is effective in reducing HbA1c levels, a major predictor of mortality in type 2 diabetes (mean decrease of −0.405, unit: %) and could improve patient safety. More studies are necessary to enhance meta-analytical power and assess the impact in other domains of care.

**Protocol registration:**

http://www.crd.york.ac.uk/PROSPERO (CRD42017070092).

## Introduction

Providing patients with access to electronic health records (EHRs) may improve quality of care by providing patients with their personal health information, and involving them as key stakeholders in the self-management of their health and disease.^1^ With the widespread use of these digital solutions, there is a growing need to evaluate their impact, in order to better understand their risks and benefits, and to inform health policies that are both patient-centred and evidence-based.

According to the Institute of Medicine (IOM), there are six domains of healthcare quality: patient-centredness, effectiveness, efficiency, safety, timeliness and equity.^2^ Patient-centred care is based on the provision of services that respect and respond to individual patients’ preferences and needs, and incorporates these aspects in clinical decisions and processes.^2 3^ Effective healthcare services result ultimately in measurable improvements in health outcomes,^4^ while ensuring the prevention of errors and adverse effects, *ie*, ensuring patient safety.^2^ Other dimensions of quality care delivery include minimising waste of resources (ie, efficiency), minimising delays in the provision of care (ie, timeliness) and avoiding differences in the provision of services to all groups of healthcare users (ie, equity).^2^

Despite the claims on the theorised benefits of providing patients with access to EHRs, there is still a considerable lack of evidence of their demonstrated impact. Though evidence suggests that these interventions improve patient satisfaction and communication^5 6^ no clear benefits were found on effectiveness.^5^ Previous studies^5 6^ were also unable to find a beneficial effect on efficiency measures, such as number of face-to-face visits and telephone appointments.

Five landmark reviews provided a comprehensive characterisation of the literature published until 2013.^5–9^ One of them^5^ included studies evaluating the impact of both paper-based and electronic records, a heterogeneity that challenges the identification of individual benefits of the digital approach. The authors of previous systematic reviews highlight the paucity of published papers, and a tendency to include small and methodologically less robust studies,^5^ with a high risk of bias.^9^ In fact, only one systematic review specifically including randomised trials was published in 2012, having found only two studies investigating the impact on effectiveness.^7^ Recent discussions around patients’ rights and data ownership have acted as strong drivers to allocate resources to interventions capitalising on EHRs with patient access.^10^ Therefore, it is plausible that the more recent literature has provided new evidence to shed light on this subject.

This work builds on the previous landmark reviews, and aims to capture recent, highest quality evidence (ie, randomised trials) in order to clarify the impact of providing patients access to EHRs. The main objective of this systematic review was to assess the impact of these interventions on the six dimensions of quality of care.

## Methods

The Preferred Reporting Items for Systematic Reviews and Meta-Analyses guidelines^11^ were followed in conducting this systematic review (online supplementary file 1). The study protocol was registered with the International Prospective Register of Systematic Reviews (PROSPERO) (CRD42017070092) and is available as an open access paper.^12^ Any differences between the protocol and review are described in online supplementary file 2.

10.1136/bmjqs-2019-010581.supp1 Supplementary data

10.1136/bmjqs-2019-010581.supp2 Supplementary data

### Search strategy

A systematic search of the literature published between 1997 and 2017 was performed on Current Index to Nursing and Allied Health Literature (CINAHL), Cochrane, Embase, Health Management and Policy Database (HMIC), Medline/PubMed and PsycINFO, using free terms and controlled vocabulary, whenever supported.^12^ The reference lists of relevant articles (including systematic reviews), and grey literature (including PROSPERO, reports of relevant stakeholder organisations (NHS Digital, AMIA, eHealth at WHO, International Society for Telemedicine and eHealth), and conference proceedings (last 5 years) of related conferences (American Medical Informatics Association, MedInfo, Medicine 2.0, Medicine X)) were also screened.

#### Study selection criteria

We included randomised trials only (see online supplementary file 2) that met the following criteria: (1) Focused on adults subjects (eg, patients, carers). (2) Included an intervention consisting of sharing EHRs with patients (either isolated or as part of a multicomponent intervention, that could include the identification of discrepancies in records, messaging systems, access to educational material, or other). (3) Had an outcome evaluating at least one of the six domains of quality of care. Studies were excluded if they (1) Included participants aged 16 years and under. (2) Had an intervention consisting of health reminders only. (3) Only reported cognitive outcomes (eg, intent) or other subjective measures only (eg, subjective perception of health and/or well-being). The detailed screening strategy is described in the study protocol.^12^

#### Data extraction

One investigator extracted information from the included studies into a standardised computer-based spreadsheet, which was reviewed by a second investigator for consistency. The data collected for each study included: name of the first author, year of publication, number of participants, participants’ characteristics and setting, date of the intervention, study duration, study design, intervention characteristics, domain of healthcare quality assessed, main outcomes (specifying if primary or secondary), effect size (means (SD) or % for every group, whenever possible; or difference between groups, if the only information available), statistical significance, overall quality score.

#### Risk of bias assessment

Risk of bias was evaluated using the Cochrane Risk of Bias Tool.^13^ Two investigators reviewed all eligible studies in order to appraise their risk of bias (ALN, LF; ALN, LL). A third investigator resolved disagreements (LL, LF). A study was considered as ‘overall low risk’ if scoring low risk for at least 50% of the criteria evaluated; otherwise, the study was considered having an ‘overall high/unclear risk’.

#### Data synthesis and meta-analysis

A narrative synthesis of results was performed by domain of quality of care (IOM framework).^2^ For the meta-analysis, continuous outcomes representing the same variable and reported in at least four studies were pooled using random effects. This was the case for HbA1c (reported as the percentage of glycated haemoglobin over the total, %), and for systolic and diastolic blood pressure (SBP and DBP, respectively; both reported in mm Hg). All effect sizes are shown as absolute difference in means (DM) (weighted mean difference (WMD)) and classified as negative when in favour of the intervention, and positive when in favour of the control. Heterogeneity was assessed using I^2^ (<30%: low; 30%–60%: moderate; 60%–90%: substantial; >90%: considerable).^13^. The presence of publication bias was evaluated by a funnel plot. Comprehensive meta-analysis V.2.3. was used for statistical analysis.

#### Sensitivity analysis and subgroup analysis

For each domain of quality, we described the proportion of studies showing beneficial effects in both ‘low risk’ and ‘unclear/high risk of bias’ groups. Sensitivity analyses were conducted, excluding high/unclear risk of bias studies (for HbA1c and SBP), and short-term interventions (lasting less than 12 months) for HbA1c. Further information is provided in online supplementary file 2.

#### Patient and public involvement

Our research question emerged from the implementation evaluation of the Care Information Exchange (https://www.careinformationexchange-nwl.nhs.uk/), a portal/EHR with patient access available to 2.4 million people in North-West London. Lay partners will be involved in summarising the research findings into lay summaries and reports.

## Results

**Figure 1:**
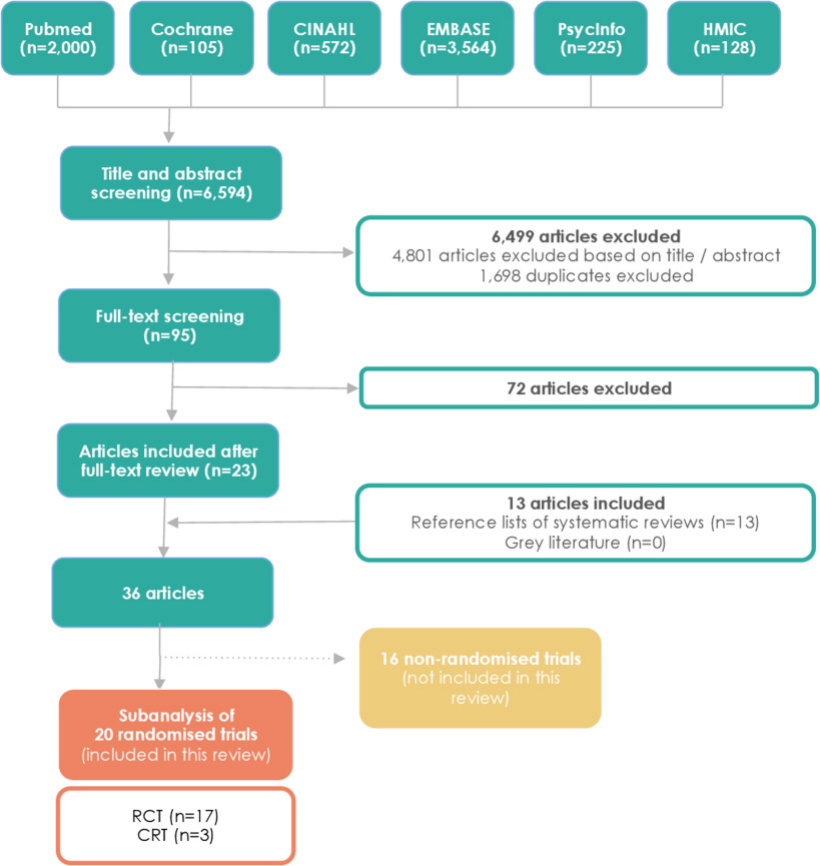
Flow diagram of included studies. CRT, cluster randomised trial; RCT, randomised controlled trial.

The database search retrieved 6594 citations (figure 1). Titles and abstracts were screened, and 1698 duplicates were excluded, as well as 4801 articles that did not meet the inclusion criteria. After the full-text screening of the remaining articles (n=95), 72 additional papers did not meet inclusion criteria and were therefore excluded. The kappa statistic measuring intercoder agreement in title and abstract screening was 0.40 (fair agreement). Screening of reference lists of systematic reviews revealed 13 additional studies that met our predefined criteria. A total of 36 papers was obtained, which included 20 randomised trials (17 randomised controlled trials (RCTs) and 3 cluster randomised trial (CRTs)).

### Description of included studies

The 20 included studies involved a total of 17 387 participants (table 1). Publication year ranged from 1999 to 2013 and study duration varied between 3 months and 32 months. Participants included had a range of health conditions, including type 2 diabetes (n=7),^14–20^ heart failure (n=2),^21 22^ arterial hypertension (n=2),^23 24^ cancer (n=1),^25^ type 1 diabetes (n=1),^26^ fertility issues (n=1)^27^ and pregnancy.^28^ Five studies included service users in general, without focusing on a specific health condition.^29–33^

**Table 1:** Characteristics of the included studies

Author, year	Study type	N* (I;C)	Date of intervention	Participants (setting)	Duration	Study design and comparison	Retention rates^†^ (%) total (I:C)	Intervention	
								EHR-sharing component	Other components of the intervention
**Chrischilles, 2013** 29	RCT	1075 (I:802; C:273)	2010–2011	General population (>65 years old) *(general public—survey to voters*)	6 M	2-arm study Standard care control group	100.0 (I:100.0; C:100.0)	Web-based health record Access to current and past medicines, allergies, health conditions, and health event tracking over time	Medication safety messages Display of general medication-use patient safety indicators
**Earnest, 2004** 21	RCT	107 (I:54; C:53)	2002	Patients with chronic heart failure (*secondary care*)	12 M	2-arm study Standard care control group	75.7 (I:70.4; C:81.1)	Web-based health record Access to medical record with clinical notes, laboratory reports, and test results, as well as information regarding heart failure	Secure messaging system
**Fonda, 2009** 14	RCT	104 (I:52; C:52)	NA	Patients with poorly controlled T2DM (*primary and secondary care*)	12 M	2-arm study Standard care control group	NA	Web-based health record Access to website which accepts electronic transmissions from blood pressure and glucose monitoring devices and displays these data in graphic and tabular form for the participant and care manager to review	Secure messaging system Web-enabled diabetes educational modules Links to other web-based diabetes resources
**Grant, 2008** 33	RCT	244 (I:126; C:118)	2005– 2007	General population (*primary care*)	12 M	2-arm study Active care control group (ie, access to a PHR to update and submit family history and health maintenance information)	64.0 (I:65.0; C:34.7)	Web-based health record Access to medications lists, glucose, blood pressure, LDL-cholesterol, preventive care and recent results and current treatment information	Secure messaging system (platform to reply to questions regarding adherence barriers and adverse effects of medication; check boxes and free text boxes within the PHR encouraged patients to enter therapy concerns and requests to address specific care limitations)
**Green,** **2008** 23	RCT	778 (I1:259; I2:261; C:258)	NA	Hypertensive patients (*primary and secondary care*)	12 M	3-arm study Standard care control group Intervention 1(I1): home BP monitoring and secure patient Web services training only Intervention 2(I2): home BP monitoring and Web training plus pharmacist care management delivered through web communications In this work, only control and intervention one were considered	93.8 (I1:94.9; I2:90.8; C:95.7)	Web-based health record Ability to view current health conditions, laboratory test results, clinic visit summaries, and lists of allergies, immunisations, and medications	Secure messaging system Ability to refill medications and make appointments
**Holbrook, 2009** 15	RCT	511 (I:253; C:258)	2002– 2003	Patients with type 2 diabetes (*primary care*)	6 M	2-arm study Standard care control group	68.7 (I:68.4; C:69.0)	Patient and primary care provider access to diabetes tracker of 13 risk factors	Targets of risk factors Personalised recommendation messages Appointment and medication reminders
**Jones,** **1999** 25	RCT	525 (I1:167; I2:178; C:180)	1997	Radiotherapy patients (*secondary care*)	3 M	3-arm study Standard care control group Intervention 1(I1): Access to general information on a computer) Intervention 2(I2): Access to personal and general information in varying order via a computer In this work only comparisons between control and I2 will be considered	83.4 (I1:76.6; I2:87.6; C:85.6)	Touch screen health record kiosk Summary of medical record, or choice between personal or general information Printout of information viewed sent to patients	Explanation about terms used were linked to in the medical records General information about cancer
**Khan,** **2010** 16	RCT	7368 (I:3856; C:3512)	NA	Patients with type 2 diabetes (*primary and secondary care*)	32 M	2-arm study Standard care control group	100.0 (I:100.0; C:100.0)	Centralised laboratory results from independent laboratories (haemoglobin A1c, cholesterol, serum creatinine, and urine protein results) accessible to patients Overdue reminders and alerts to patients with elevated test results	Generation of flow sheets with laboratory results, reminders of overdue laboratory tests, and summary population reports for providers
**Krist,** **2012** 32	RCT	4500 (I:2250: C:2250)	2008– 2009	General population (*primary care*)	16 M	2-arm study Standard care control group	NA	Access to relevant details in the patient’s history (prior laboratory test values and dates)	Preventive services recommendations based on EHR data Links to relevant informational material and decision aids
**McCarrier, 2009** 26	RCT	78 (I:42; C:36)	2005– 2006	Patients with type 1 diabetes (*primary care*)	12 M	2-arm study Standard care control group	83.3 (I:85.7; C:80.6)	Web-based health record Access to entire EHR with clinical encounters, physician notes, and test results	Blood glucose readings uploaded by patients Medication, nutrition, and exercise data can be registered by both patients and case managers Generations of action plans for self-efficacy and self-management support Educational information on diabetes
**McMahon, 2005** 17	RCT	104 (I:52; C:52)	2004	Patients with type 2 diabetes patients (*both primary and secondary care*)	12 M	2-arm study Standard care control group	75.9 (I:75.0; C:76.9)	Web-based care-management site with value upload for blood pressure and glucose monitoring devices Graphical and tabular view of measurements provided for patients and HCPs	Half-day self-management training on diabetes Computer training and support available to intervention participants Messaging with care manager *via* site Educational material
**Nagykaldi, 2012** 31	CRT	384 (I:NA; C:NA)	NA	General population (*primary care*)	12 M	2-arm study Standard care control group	68.5 (I:NA; C:NA)	Web-based patient portal with option to manage health information and download a personal health record	Personalised wellness plan, prevention and longitudinal health information available
**Quinn, 2008** 19	RCT	26 (I:13; C:13)	2006	Patients with type 2 diabetes patients (*primary care*)	3 M	2-arm study Standard care control group	NA	Blood glucose meter value sent directly to the patient’s mobile phone Real-time feedback on blood glucose levels Display of medications	Educational information
**Ralston, 2009** 18	RCT	83 (I:42; C:41)	2002– 2004	Patients with type 2 diabetes (*secondary care*)	12 M	2-arm study Standard care control group	90.3 (I:92.95; C:87.8)	Web-based EHR access Feedback on blood glucose measurements	Messaging system for patients and staff Educational information (exercise, diet and medication)
**Ross,** **2004** 22	RCT	107 (I:54; C:53)	2001	Patients with heart failure (*secondary care*)	12 M	2-arm study Standard care control group	75.7 (I:81.1; C:70.3)	Web-based EHR access practice	Messaging system for patients and staff Educational information
**Schnipper, 2012** 30	CRT	541 (I:267; C:274)	2005– 2007	General population (*primary care*)	NA	2-arm study Active care control group (ie, patients received a different EHR-linked intervention)	74.3% (I:100.0% C:49.3%)	Web-based medication module linked to EHR	Ability to request appointments and referrals Communication with their physician via secure email Prescription renewals and access a health information library
**Shaw,** **2008** 28	RCT	193 (I:97; C:96)	2004– 2006	Maternity centre (*primary care*)	NA	2-arm study Active care control group (ie, patients received access to the same website but with links to general pregnancy health information alone)	54.9 (I:64.9; C:44.8)	Web-based access to antenatal health record	Access to general pregnancy health information website for control and intervention groups
**Tang,** **2013** 20	RCT	415 (I:202; C:213)	2008– 2009	Patients with type 2 diabetes patients (*both primary and secondary care*)	12 M	2-arm study Standard care control group	91.3 I:92.0; C:90.6	Web-based patient portal access to EHR Automatic upload of blood glucose values with visual feedback; Personalised diabetes summary; nutrition, exercise, and insulin records	Online messaging with HCPs Advice and medication management from HCPs Personalised e-educational materials
**Tuil,** **2007** 27	RCT	244 (I:122; C:122)	2004	Patients undergoing IVF or ICSI (*secondary care*)	NA	2-arm study Standard care control group	73.7 (I:83.6; C:63.9)	Web-based EHR Personal and general information regarding treatment	Communication with other patients and HCPs
**Wagner, 2012** 24	CRT	443 (I:194; C:252)	NA	Patients with hypertension (*both primary and secondary care*)	12 M	2-arm study Standard care control group	71.9 (I:61.8; C:75.8)	Web-based EHR Patients could view problem and medication lists, information on allergies and immunisation	Messaging function, educational materials, medication interaction checking, health measurement tracking, and health diaries

*Total number of participants randomised for each study.

†Retention rates were calculated as the proportion of patients randomised in each study that completed follow-up.

BP, blood pressure; C, Control group; CRT, cluster randomised trial; EHR, electronic health records; HCP, healthcare professionals; I, Intervention group; ICSI, intracytoplasmic sperm injection; IVF, in vitro fertilisation; LDL, low-density lipoprotein; M, months; NA, information not available; PHR, personal health record; RCT, randomised controlled trial; T2DM, type 2 diabetes mellitus.

### Summary of risk of bias assessment

**Figure 2:**
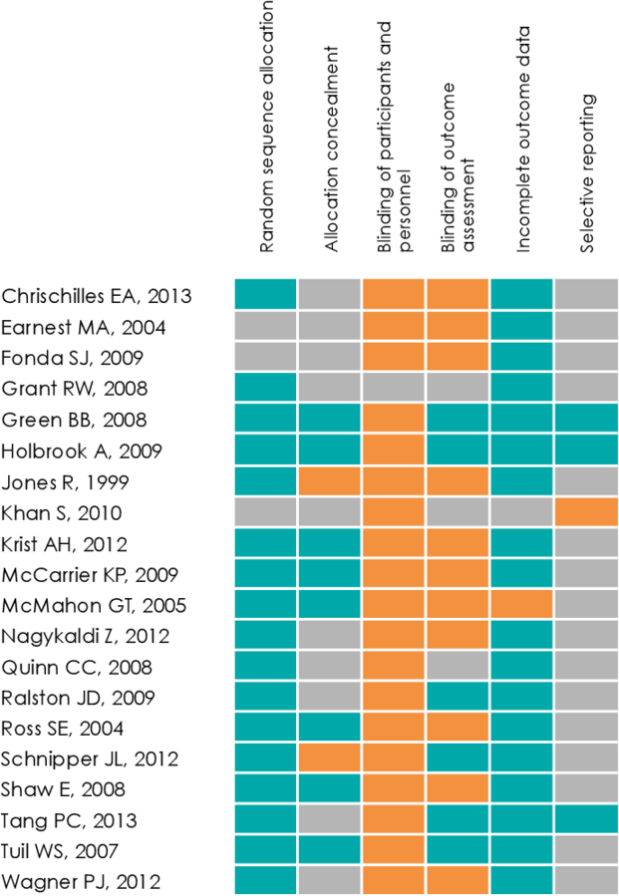
Risk of bias assessment cells were colour-coded in orange for high risk of bias, in green for low risk of bias and in grey if risk of bias was unclear.

Overall, 50% of the studies included (n=10) were considered good quality, scoring low risk in at least half of the domains evaluated in the risk of bias assessment (figure 2).^15 18 20 22 23 26–28 30 32^. Four studies stood out with an overall low risk of bias for most of the domains evaluated.^15 20 23 27^ Due to the nature of the intervention, most studies scored a high risk of bias regarding blinding of participants and personnel; one study showed unclear risk.^33^ Blinding of the outcome assessment also showed a high risk of bias in several studies.^14 17 21 22 24–26 28 29 31 32^ Only three studies^15 20 23^ provided information on trial protocol registration.

### Interventions and retention rates

Although all interventions provided participants with web-based access to EHRs, the content made available varied greatly (table 1). Content available to participants included access to previous medical history and risk factors,^15 20 24 25 28 29 31^ test results,^14 16–19 21 26 31–33^ medication lists,^23 24 30 31 33^ list of allergies,^23 24^ current health conditions,^23 31^ and clinical encounters and physician notes.^26 31^ One study specifically mentioned the existence of a functionality to download EHR data.^31^ In all studies, the patient access to EHRs was part of a complex intervention with other components. Intervention components included educational materials,^14 18–20 22 24–26 28 30–32^ generation of personalised action plans/messages,^15 26 31 32^ self-management training,^17^ and medication and appointment reminders.^15 16^ Twelve studies included secure messaging systems.^14 17 20–24 27 29–31 33^ Two studies provided incentives (either financial,^29^ or use of the portal after the study),^22^ and one explicitly mentioned that no incentives were provided.^18^ Retention rates were calculated as the proportion of randomised patients in each study that completed follow-up. Three studies did not provide enough information to adequately calculate retention rates (total and per arm).^14 19 32^ Among the other studies, only one^28^ had a retention rate below 60%.

### Comparisons

In most studies, the comparator was usual care (ie, no patient access to EHRs).^14–22 24 26 27 29 31 32^ In three studies, the comparisons were active controls.^28 30 33^ Two studies comprised three arms,^23 25^ which are described in further detail in table 1.

### Outcomes

Most papers assessed outcomes covering more than one domain (median=2). The domain most frequently assessed was effectiveness (n=14), and the least frequently evaluated were timeliness and equity (n=0). Patient-centredness, safety and efficiency were evaluated, respectively, in 11, 4 and 5 studies. A detailed overview of the outcomes evaluated is provided in online supplementary file 3.

10.1136/bmjqs-2019-010581.supp3 Supplementary data

#### Patient-centredness

Eleven studies evaluated the impact of sharing EHRs with patients on patient-centredness, including CRTs^24 30 31^ and eight RCTs.^19–22 25–28^ While six studies found a beneficial impact in at least one patient-centredness outcome,^20 24–26 30 31^ it is important to note that the exact measure of patient-centredness varied considerably across studies. Although patient satisfaction improved in two studies^20 25^ (46% vs 40%, p=0.04% and 27.7% vs 24.5%, p<0.0001, respectively), two other failed to show a significant effect.^22 27^ One study^31^ showed an increase in patient activation, as measured by the Patient Activation Measure^34^ (47 vs 45, p=0.0014), but these results were not replicated in a similar study.^24^ Self-efficacy scores improved in one study^26^ using the Diabetes Empowerment Scale^35^ (+0.14 vs −0.16, p=0.04), but no differences were found in two other studies^22 27^ using the Kansas City Cardiomyopathy Questionnaire (KCCQ) and the General Self-Efficacy Scale.^36^ Patient empowerment was accessed by the Patient Empowerment Scale^37^ in two studies^21 24^ but a significant improvement in mean scores was found only in one (41.2 vs 40.1, p=0.019).^24^ Three studies evaluated health literacy (ie, patients acknowledging to have learnt something new),^19 25 28^ but only one found the intervention to be beneficial (96% vs 74%, p=0.02). Six out of 11 studies (54.5%) scored an overall low risk of bias. The proportion of studies showing a significant positive effect for at least one of the outcomes evaluated was 50% in low risk of bias studies, and 80% in the remaining studies.

#### Effectiveness

A total of 14 studies appraised the impact of providing patients with access to EHRs on effectiveness, including 2 CRTs^24 31^ and 12 RCTs.^14 15 17–20 22 23 25 26 32 33^ Ten out of 14 studies (71.4%) demonstrated a positive impact on effectiveness-related outcomes.^15 17–20 22 23 25 31 32^ These studies evaluated the impact on a wide range of health conditions, including depression and anxiety,^25^ heart failure,^22^ cardiovascular risk (Framingham Score),^20^ obesity,^15 23^ smoking status,^15^ adherence to preventive services^31 32^ dyslipidaemia,^17 18 20 24 33^ diabetes^14 15 17–20 26 33^ and hypertension.^15 17 18 20 23 24 33^ In one study using the Hospital Anxiety and Depression Scale,^38^ patient access to EHRs did not change patients’ depression scores, and patients in the general computer information group were more anxious than the ones accessing personal records (DM=+18%, 95% CI 3.7 to 26.5, p=0.001).^25^ One study found a dramatic improvement in symptom stability scores, assessed by the KCCQ (DM:+17, 95% CI 9 to 29, p<0.001).^22^ Two studies found an improvement in LDL-cholesterol levels.^17 20^ No significant changes were observed on triglycerides,^17^ high-density lipoprotein (HDL)-cholesterol,^17^ total cholesterol,^18^ body weight,^15 23^ smoking status^15^ or total cardiovascular risk.^20^ Adherence to preventive services improved in the two studies evaluating this aspect^31 32^ (ie, use of low-dose aspirin (84.4% vs 67.6%, p<0.0001), complete immunisation (95.5% vs 87.2%, p=0.044), and uptake of cancer screening (increases ranging from 10.3% to +14.3%, all p<0.05)). While two studies specifically evaluated adherence to pneumococcal immunisation,^31 32^ only one found a beneficial effect.^31^

Seven out of 14 studies scored an overall low risk of bias (50.0%). The proportion of studies showing a positive effect was 85.7% in the low risk of bias group, and 57.1% in the remaining studies.

##### Meta-analysis

**Figure 3:**
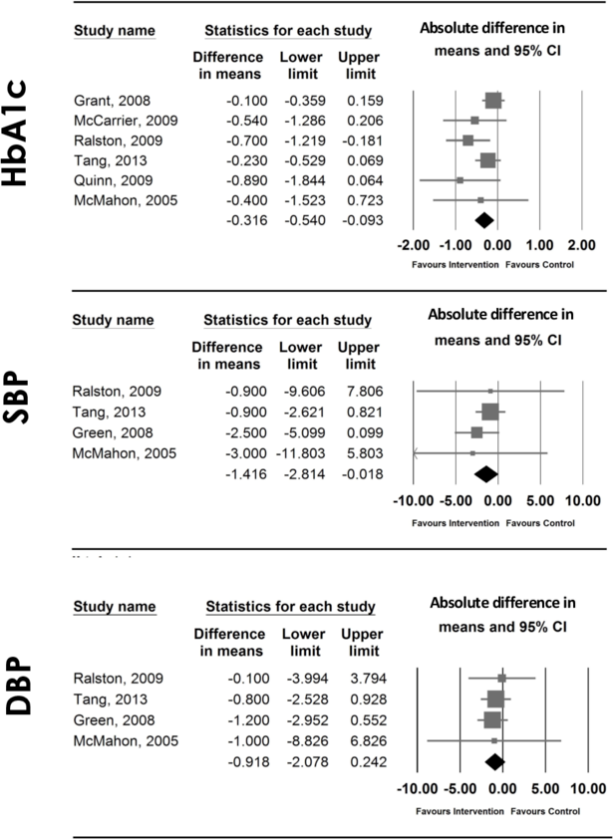
Forest plots of effect sizes and 95% CIs representing the effect of interventions providing patients access to EHRs in HbA1c, SBP and DBP, using a random-effects model. The area of each square is proportional to the study's size, and therefore to its weight in the meta-analysis. For each study, CIs are represented by horizontal lines; a vertical line representing no effect is also plotted. The meta-analysed measure of effect is plotted as a diamond, the lateral points of which indicate CIs for this estimate. DBP, diastolic blood pressure; EHRs, electronic health records; HbA1c, glycated haemoglobin, SBP, systolic blood pressure.

Data from RCTs evaluating HbA1c and SBP were pooled together, and the respective meta-analyses performed. The six studies evaluating HbA1c^17–20 26 33^ comprised 950 participants, from which 894 completed follow-ups. Meta-analyses showed a beneficial effect in effectiveness by reducing HbA1c (unit, %; WMD= −0.316; 95% CI −0.540 to −0.093, p=0.005, I^2^=0%) (figure 3), which remained significant in sensitivity analyses for low risk of bias studies (WMD= −0.405; 95% CI −0.711 to −0.099) (online supplementary figure 1), and long-term interventions only (WMD= −0.272; 95% CI −0.482 to −0.062) (online supplementary figure 2). It is important to note that the study showing a high risk of bias,^19^ was also the one showing the smallest study sample. The funnel plot indicates asymmetry (online supplementary figure 3), suggesting potential publication bias.

10.1136/bmjqs-2019-010581.supp4 Supplementary data

10.1136/bmjqs-2019-010581.supp5 Supplementary data

10.1136/bmjqs-2019-010581.supp6 Supplementary data

The four studies evaluating the impact on blood pressure^17 18 20 23^ (comprising 1308 participants, of which 1021 completed follow-ups) were pooled in a meta-analysis, and showed a significant beneficial effect in SBP (unit: mm Hg; WMD=−1.416; 95% CI −2.814 to −0.018, p=0.047, I^2^=0%) (figure 3). However, significance was lost after removing the high/unclear risk of bias study^17^ (WMD=−1.375; 95% CI −2.791 to 0.041) (online supplementary figure 1). No significant effect was found in DBP in the meta-analysis (unit: mm Hg; WMD=−0.918; 95% CI −2.078 to 0.242, p=0.121, I^2^=0%) (figure 3), nor in the sensitivity analysis for low risk of bias studies only (WMD=−0.916; 95% CI −2.089 to 0.257) (online supplementary figure 1). The funnel plots appear symmetrical for SBP and DBP (online supplementary figures 4 and 5), indicating a similar proportion of studies in each direction of the effect size.

10.1136/bmjqs-2019-010581.supp7 Supplementary data

10.1136/bmjqs-2019-010581.supp8 Supplementary data

#### Safety

All studies^19 22 29 30^ showed a beneficial effect for at least one of the outcomes evaluated (online supplementary file 3). Two studies evaluated adherence, including general adherence to medical regimens^22^ (using the General Adherence Scale from the Medical Outcomes Study (MOS)^39^ and medication adherence.^22 29^ General adherence (MOS Scores) improved with the intervention^22^ (+2.3, 95% CI −3.7 to 8.3, p=0.01), but no significant changes were found in adherence to medication.^22 29^ A beneficial effect was observed in all studies evaluating medication safety,^19 29 30^ including a higher likelihood of reporting discrepancies (53% vs 24%, p<0.01),^29^ to change medications^19 29^ (88.3% vs 67.2%, p<0.01; and 84% vs 23%, p=0.002, respectively), and a resulting slightly lower proportion of patients with medication discrepancies (29% vs 30%, p=0.01).^30^ Two out of four studies scored an overall low risk of bias, and the proportion of studies showing a positive effect was the same in both risk groups (100.0%).

#### Efficiency

The impact of providing patients with access to EHRs was assessed in five studies.^16 18 22 24 31^ As less than four studies assessed the same construct, meta-analysis was not performed, and a descriptive analysis is provided. Number of hospitalisations per subject was lower in one study (0.17 vs 0.20, p=0.01),^16^ while total number remained unchanged in another (22 vs 21, p=1.00).^22^ Length of stay (in days) did not change in two studies (+0.2 vs –0.3, and 0.42 vs 0.34, respectively),^18 24^ but was shorter in another (0.99 vs 1.1, p<0.01).^16^ In the three studies evaluating the number of emergency visits, total numbers were either reduced,^16^ increased^22^ or remained unchanged.^24^ Number of primary care visits was lower in one study (2.9 vs 4.3, p<0.0001),^31^ but no changes were observed in another (0.0 vs –0.2).^18^ Two out of five studies scored an overall low risk of bias, and the proportion of studies showing a positive effect was 50.0% and 66.7% in low-risk and high/unclear-risk groups, respectively.

#### Timeliness and equity

While none of the studies assessed either timeliness or equity as primary outcome, three studies^21 24 32^ evaluated the predictors of usage of EHRs by patients. Earnest *et al* 21 did not find any associations between usage and race, symptom scores or number of visits; two studies found significant associations between usage and higher education,^32^ number of illnesses,^32^ younger age,^24^ clinic attended by the patient^24^ self-reported computer skills,^24^ and higher number of internet-use items.^24^

## Discussion

### Key findings in context of published literature

This work systematically appraised the impact of EHRs with patient access across the six domains of quality of care as defined by the IOM:^2^ patient-centredness, effectiveness, efficiency, safety, timeliness and equity.

Regarding patient-centredness, results were inconsistent. More than half of the studies included in this domain showed a significant positive effect for at least one outcome, but no clear effect was found for specific outcomes, such as patient satisfaction, patient activation, self-efficacy, patient empowerment or health literacy. These results are line with previous studies^5 6 8^ that found mixed evidence about the impact in patient-centred outcomes. While providing patients access to EHRs is envisaged as a key strategy to deliver patient-centred care, the diversity of outcomes evaluated, and scales and tools used, hinders pooling of results and the use of meta-analytical approaches. It is critical, therefore, to identify and standardise measures and constructs to evaluate patient-centredness, to allow the application of meta-analytical methods in this domain.

A few studies included showed a positive impact in effectiveness in a range of outcomes (ie, anxiety, cardiac symptoms, LDL-cholesterol), but no significant improvements were found for triglycerides, HDL-cholesterol, total cholesterol, body weight, smoking status or total cardiovascular risk. Two additional studies not captured by our search also suggest that providing patients access to EHRs may improve glaucoma control^40^ and quality of life in patients with asthma.^41^ A positive effect was also found in adherence to several preventive services (ie, use of low-dose aspirin, cancer screening), an approach that can be particularly relevant in the context of cancer screening, where higher expected adherence rates have the potential to reduce cancer incidence and mortality.^42^ However, the number of studies published per outcome is limited, and further research is needed to increase meta-analytical power and explore the size and impact of the potential effect in specific health conditions.

Our meta-analysis showed a beneficial effect on HbA1c reduction, which remained significant after removing low/unclear-risk studies, or studies in which the intervention lasted less than 12 months. In 2013, Goldzweig *et al* identified several examples of improved outcomes for patients with chronic diseases (including hypertension and diabetes).^8^ In 2012, Ammenwerth *et al* ^7^ performed a systematic review of studies published between 1990 and 2011 and concluded that there was insufficient evidence to document a beneficial effect in effectiveness in patients with access to EHRs. However, by then only two studies (out of the four included in the review) investigated the effect on health outcomes. Our meta-analysis demonstrates a mean reduction in absolute values of HbA1c of 0.316% (95% CI −0.540% to −0.093%), with a low heterogeneity (I^2^=0.0) reflecting the specificity of our inclusion criteria. These results have important clinical implications, since an absolute reduction of 1 point on HbA1c levels (expressed in the same unit considered in our meta-analysis) is associated with a significant reduction of deaths related to diabetes (−21%), myocardial infarction (−14%) and microvascular complications (−37%).^43^ Visual inspection of the funnel plot suggests a potential publication bias, with studies with a lower precision (higher SE) reporting a greater beneficial effect. However, the meta-analysed effect remained significant after removing the study that stood out with a smallest sample size.^19^

Although our meta-analysis found a beneficial effect in SBP, statistical significance was lost in sensitivity analysis for low risk of bias studies only; no significant effect was found in DBP. It must be noted, however, that the number of studies included is low, and further evidence is needed to establish robust conclusions.

For the efficiency domain, most studies included found either no change, or a reduction of healthcare usage (in primary care visits,^31^ or inpatient or emergency contacts).^16^ Ammenwerth *et al*,^7^ have also previously suggested a significant reduction in office visit rates. Further studies are required to clarify the impact on this dimension and pave the way to meta-analytical approaches that can provide further insights on the effect size in the various dimensions of healthcare usage.

Our work suggests that the intervention improves general adherence, but not medication adherence—however, a strong body of evidence showed a positive effect in medication safety. A previous study has suggested that patients find this approach valuable, and reported either unchanged or improved relationships with their clinician when using it.^44^ Further studies should further explore patients’ willingness and ability to report errors in their records, and also which specific groups are most likely to benefit. These results are in line with the findings of Mould *et al*, de Lusignan *et al* and Ammenwerth *et al*, who previously suggested that these digital solutions positively impacted patient safety.^6 7 9^

Finally, we found no studies specifically focusing on the impact on timeliness or equity. Uptake of portals may differ by patient-specific factors, with lower use by racial and ethnic minorities, patients with lower education level or literacy, thus leading to digital-led health inequities.^8^ Davis Giardina *et al* 5 reported that, up to 2012, no studies had assessed any of these domains. Eight years later, these aspects remain unexplored.

### Strengths and limitations

Five landmark reviews have been published to date evaluating the impact of EHRs with patient access on different aspects of quality of care.^5–9^ Only one systematic review had focused on randomised trials, having found two studies investigating the impact on effectiveness.^7^

Our systematic review included studies published between 1997 and 2017 and retrieved a total of 20 randomised trials. This study has several strengths: a predefined, openly available protocol was followed^12^ (with any changes described in online supplementary file 2); only randomised trials were included; focused exclusively on EHRs; and impact was assessed in all domains of quality of care, with meta-analysis performed whenever possible.

Only half of the studies included had an overall low risk of bias score. A possible approach to improve blinding in web-based interventions, or to test the impact of specific components, could be using A/B testing, a technique used for website optimisation that compares variation against a standard experience, and determines which variant is more effective.^45^

## Conclusion

Our results suggest that providing patients with access to EHRs can improve patient safety and effectiveness. More methodologically robust studies are necessary to increase the strength of these conclusions, and to enhance meta-analytical power. For EHRs with patient access to be broadly used, it is important to focus on interventions that enhance adoption and measure usage, and issues of equity in both aspects need to be addressed by policy makers when implementing such programmes.^46^
