# Supplementary material for: Impact of providing patients access to electronic health records on quality and safety of care: a systematic review and meta-analysis
Source: BMJ Qual Saf. 2020 Jun 12;29(12):1019–32. doi: 10.1136/bmjqs-2019-010581 (PMC7785164; doi:10.1136/bmjqs-2019-010581)
Supplement: Supplementary data [file bmjqs-2019-010581supp002.pdf]

Supplementary File 2: Differences between protocol and review

Inclusion criteria

The search strategy performed, as described in the protocol paper, included both randomised and non-randomised trials. As we were unsure of the amount of evidence on the subject, we decided to capture all recent evidence at the first stage (outlined in Figure 1). Since the search retrieved a high number of randomised trials (n=20) and had enough evidence, we decided to include only the more robust, highest-quality studies, i.e. randomised trials. We acknowledge that this was a deviation to the original protocol, but we believe it enhanced the robustness of the present work. A full list of the trials retrieved in the search is available on request from the corresponding author.

Sensitivity analyses per duration of the intervention

In the protocol, we planned to perform sensitivity analysis by the time of duration (short-term, medium-term, and long-term). However, these were not always possible due to the characteristics of the studies retrieved, as explained below.

- For HbA1c, sensitivity analyses were performed for low-risk of bias studies only, and for studies with a long duration only (≥12 duration).
- For SBP and DBP, a sensitivity analysis including only low-risk of bias studies was performed. Sensitivity analysis by duration of intervention was not possible in this case, since all studies had the same duration (12M)
