# Supplementary material for: Impact of providing patients access to electronic health records on quality and safety of care: a systematic review and meta-analysis
Source: BMJ Qual Saf. 2020 Jun 12;29(12):1019–32. doi: 10.1136/bmjqs-2019-010581 (PMC7785164; doi:10.1136/bmjqs-2019-010581)
Supplement: Supplementary data [file bmjqs-2019-010581supp003.pdf]

Supplementary File 3. Summary of outcomes evaluated in the included studies

Author, date	Outcomes	Effect size	*p*-val	Quality score
**PATIENT-CENTREDNESS**				
Jones R, 1999[25]	Primary:<br>• Higher proportion of patients with a high satisfaction score, *n(%)*<br>• Increased health literacy (*i.e.* having learnt something new), *n(%)*	 C: 58(40); I: 68 (46)<br>C: 74 (49); I: 96 (62)	 **p=0.04**<br>**p=0.02**	◆
McCarrier KP, 2009[26]	• Increased psychosocial self-efficacy (Diabetes Empowerment Tool), *mean (SD)*	C: -0.16(0.62); I: 0.14 (0.62)	**p=0.04**	◆
Nagykaldi Z, 2012[31]	Primary<br>• Increased patient activation scores (Patient Activation Measure - PAM), *mean*<br>• Increased participants' perception of patient-centeredness of care scores, *mean*	 C: 45; I; 47<br>C: -0.43; I: 0.32	 **p=0.0014**<br>**p=0.037**	◆
Schnipper JL, 2012[30]	Secondary<br>• Increased proportion of patients telling their doctor about any medication-related problems or new symptoms, %<br>• Increased proportion of patients telling their doctor about all prescribed medications, %	 C: 87.1; I: 97.9<br>C: 69.8; I: 74.8	 **p<0.001**<br>**p=0.048**	◆
Tang PC, 2013[20]	• Increased overall treatment satisfaction scores (Diabetes Treatment Satisfaction Questionnaire - DTSQ), *mean (SD)*	C: 24.5(7.4); I: 27.7(6.1)	**p<0.001**	◆
Wagner PJ, 2012[24]	• No significant differences in patient activation (PAM), *mean*<br>• Increased Patient Empowerment Scale (PES) scores, *mean*	C: 69.1; I: 71.4<br>C: 40.1; I: 41.2	p=0.490<br>**p=0.019**	◆
Earnest MA, 2004[21]	Primary<br>• No differences developed between the intervention and the control group in PES scores	NA	p>0.05	◆
Tuil WS, 2007[27]	Primary<br>• No significant changes in scores of self-efficacy (General Self-Efficacy Scale) post-test<br>○ Involvement in the decision process (male), *mean (SD)*<br>○ Involvement in the decision process (female), *mean (SD)*<br>○ General self-efficacy (male), *mean (SD)*<br>○ General self-efficacy (female), *mean (SD)*<br>○ Specific self-efficacy (male), *mean (SD)*<br>○ Specific self-efficacy (female), *mean (SD)*<br>○ Subjective knowledge (male), *mean (SD)*<br>○ Subjective knowledge (female), *mean (SD)*<br>○ Objective knowledge (male), *mean (SD)*<br>○ Objective knowledge (female), *mean (SD)*<br>• No significant change in patient satisfaction post-test (male), *mean (SD)*<br>• No significant change in patient satisfaction post-test (female), *mean (SD)*	C:48.0(5.2); I:48.5(4.4)<br>C: 48.7(5.2); I: 49.0(5.1)<br>C: 3.3(0.4); I: 3.3.(0.4)<br>C:3.3(0.4); I: 3.2(0.3)<br>C:14.5(2.5); I: 14.9(2.7)<br>C: 14.9(2.7); I:14.6(2.9)<br>C:7.1(1.5); I: 7.4(1.3)<br>C: 7.3(1.2); I: 7.3(1.3)<br>C: 6.0(1.4); I: 6.1(1.3)<br>C: 6.0(1.7); I: 6.0(1.5)<br>C: 123.2(11.2); I: 121.7(11.3)<br>C: 119.8(10.2); I: 120.7(8.6)	p=0.791<br>p=0.794<br>p=0.859<br>p=0.505<br>p=0.943<br>p=0.646<br>p=0.472<br>p=0.510<br>p=0.789<br>p=0.612<br>0.832<br>0.837	◆

Shaw E, 2008[28]	Primary:<br>• No significant changes in factors of health literacy (response scores)<br>○ Learning something new, *mean (SD)*<br>○ Learning about risks<br>○ Learning to understand tests<br>• No significant changes on patient empowerment (*i.e.* ability to make decisions)	 <br>C: 1.9(0.8); I: 1.8(0.9)<br>C: 1.8(0.8); I: 1.9 (0.8)<br>C: 1.6(0.8); I: 1.7(0.7)<br>C: 2.4(1.2); I:2.1(0.9)	 <br>p=0.67<br>p=0.46<br>p=0.57<br>p=0.26	◆
Ross SE, 2004[22]	Primary<br>• No significant change in self-efficacy (Kansas City Cardiomyopathy Questionnaire - KCCQ), *mean difference between groups (95CI)*<br><br>Secondary<br>• No significant change in patient satisfaction	+6 [-1 to 11]<br><br>+0.2 [-0.2 to 0.5]	p=0.08<br><br>p=0.07	◆
Quinn CC, 2008[19]	Secondary:<br>• No significant differences in self-reported health literacy (*i.e.* knowledge of food choices), *%*	C: 50.0; I: 90.0	**p=0.062**	◆

EFFECTIVENESS

Green BB, 2008[23]	Primary:<br>• Decreased systolic blood pressure values, *adjusted mean change (95CI)*<br>• No difference on diastolic blood pressure values, *adjusted mean change (95CI)*<br>• No difference on the proportion of patients with controlled BP, *adjusted RR (95CI)*<br><br>Secondary:<br>• Lower number of antihypertensive medication classes, *mean (SD)*<br>• No difference in body mass index values, *mean (SD)*	C: -5.3[-7.1 to-3.5]; I: -8.2 [-10.0 to -6.4]<br>C: -3.5[-4.5 to -2.5]; I: -4.4[5.4 to -3.4]<br>C: 1[Reference]; I: 1.22 [0.95 to1.56]<br><br>C: 1.69 (0.91); I: 1.94 (0.91)<br>C: 32.5 (6.5); I: 32.5 (7.0)	**p=0.02**<br>p=0.21<br>p=0.20<br><br>**p<0.01**<br>p>0.05	◆
Holbrook A, 2009[15]	Primary:<br>• Improved number of patients with improvement for total composite score in diabetes care processes, *n (%)*<br>• Patient with improvement of ≥ 3 points on total composite score, *n (%)*<br><br>Secondary:<br>• Decreased systolic blood pressure values, *mean difference between groups (95CI)*<br>• Decreased diastolic blood pressure values, *mean difference between groups (95CI)*<br>• Decreased HbA1c values, *mean difference between groups (95CI)*<br>• No significant differences in LDL-cholesterol levels, *mean difference between groups (95CI)*<br>• No differences in albuminuria, *mean difference between groups (95CI)*<br>• No differences in body mass index values, *mean difference between groups (95CI)*<br>• No differences in physical activity (min/week), *mean difference between groups (95CI)*<br>• No differences in the prevalence of neuropathy, *mean difference between groups (95CI)*<br>No differences in smoking status, *mean difference between groups (95CI)*	C: 110(42.6); I: 156 (61.7)<br>C: 46 (17.8); I: 88 (34.8)<br><br>–3.95 [–7.64 to - 0.26]<br>–2.38 [–4.60 to 0.17]<br>–0.20 [–0.38 to –0.02]<br>–0.002 [–0.14 to 0.14]<br>+0.65 [–1.11to 2.41]<br>+0.02 [–1.24 to 1.28]<br>+5.18 [–43.50 to 53.86]<br>0.01 [–0.08 to 0.10]<br>–0.02 [–0.09 to 0.04]	**p<0.001**<br>**p<0.001**<br><br>**p=0.036**<br>**p=0.049**<br>**p=0.029**<br>p>0.05<br>p>0.05<br>p>0.05<br>p>0.05<br>p>0.05<br>p>0.05	◆
McMahon GT, 2005[17]	Primary:<br>• Greater reduction in HbA1c values, *mean (SD)*<br>• Greater reduction in systolic blood pressure values, *mean (SD)*<br>• No significant changes in diastolic blood pressure, *mean (SD)*	C: -1.2(1.4); I: -1.6(1.4)<br>C: -7(21); I: -10(17)<br>C: -6(11);I: -5(13)	**p<0.05**<br>**p<0.01**<br>p=0.058	◆

	Secondary:<br>• Greater reduction in triglycerides values, *mean (SD)*<br>• Significant increase in HDL-cholesterol values, *mean (SD)*<br>• No changes in LDL-cholesterol levels, *mean (SD)*	 C: -2(60); I: -38(99)<br>C: 1(6); I: 3(6)<br>C:-5(11); I:-6(12)	 **p<0.01**<br>**p<0.05**<br>p>0.05	
Quinn CC, 2008[19]	Primary<br>• Greater reduction in A1c values, mean	 C: -0.68; I: -2.03	 **p < 0.04**	◆
Ralston JD, 2009[18]	Primary<br>• Reduction in mean HbA1c levels, *mean difference between groups*<br>• Higher change on % of patients with HbA1c levels <7%, *mean difference between groups*<br><br>Secondary<br>• No significant changes in systolic blood pressure, *mean difference between groups*<br>• No significant changes in diastolic blood pressure, *mean difference between groups*<br>• No significant changes in total cholesterol levels, *mean difference between group*	 -1.1<br>-22<br><br><br>-0.9<br>+0.1<br>+7.6	 **p<0.01**<br>**p<0.03**<br><br><br>p=0.84<br>p=0.96<br>p=0.38	◆
Tang PC, 2013[20]	Primary<br>• Decreased HbA1c levels at 6 M, *mean (SD)*<br>• No significant changes on HbA1c levels at 12 M, *mean (SD)*<br><br>Secondary<br>• Decreased LDL cholesterol levels at 12 M, *mean (SD)*<br>• No significant differences in weight (in pounds), at 12 M, *mean (SD)*<br>• No significant differences in systolic blood pressure (mmHg) at 12 M, *mean (SD)*<br>• No significant differences in diastolic blood pressure (at 12 mo, *p*=0.374), *mean (SD)*<br>• No significant differences in cardiovascular risk (Framingham score) at 12 M, *mean (SD)*	 C: 8.62(1.94), I: 7.92 (1.39)<br>C: 8.33(1.81); I: 8.10(1.68)<br><br><br>C: 98.4(32.4); I: 88.9 (33.5)<br>C: 215.7(51.0); I: 218.8(52.9)<br>C: 120.8 (11.5); I: 119.9 (11.4)<br>C: 72.5(8.3); I: 71.7(8.9)<br>C: 5.2(5.7); I: 4.9(5.4)	 **p<0.001**<br>p=0.133<br><br><br>**p=0.001**<br>p=0.232<br>p=0.306<br>p=0.374<br>p=0.051	◆
Jones R, 1999[25]	Primary:<br>• Decreased % of patients with anxiety, *mean difference between groups (95CI)*	 18 [3.7; 26.5]	 **p=0.001**	◆
Ross SE, 2004[22]	Primary:<br>• Increased symptom stability (*p*<0.01), *mean difference between groups (95CI)*<br>• No changes in quality of life, *mean difference between groups (95CI)*<br>• No changes in functional status (*p*=0.31), *mean difference between groups (95CI)*<br>• No changes in physical limitations physical limitations, *mean difference between groups (95CI)*<br>• No changes in mortality, *n(%)*	 +17 [4 to 29]<br>+2 [-7 to 11]<br>-3 [-11 to 3]<br>-4 [-12 to 3]<br>C: 6(11); I: 6(11)	 **p<0.01**<br>p=0.63<br>p=0.31<br>p=0.26<br>p=1.00	◆
McCarrier KP, 2009[26]	Primary:<br>• No significant differences in HbA1c change in scores, *mean (SD)*	 C: +0.11(1.4); I: - 0.37(1.3)	 p=0.160	◆
Grant RW, 2008[33]	Primary<br>• No significant differences in mean HbA1c levels between arms, *mean*<br>• No significant differences in blood pressure between arms<br>• No significant changes in LDL-cholesterol between arms	 C: 7.1; I:7.2<br>NA<br>NA	 p=0.450<br>p>0.05<br>p>0.05	◆
Fonda SJ, 2009[14]	Primary<br>• No significant difference in Problem Areas in Diabetes (PAID) scores, *difference in means*	 -3.1	 p=0.300	◆

Wagner PJ, 2012[24]	Primary<br>• No significant differences in systolic pressure, *mean*<br>• No significant differences in systolic pressure, *mean*<br><br>Secondary<br>• No significant differences in HDL-cholesterol levels, *mean*<br>• No significant differences in LDL-cholesterol levels, *mean*<br>• No significant differences in triglycerides levels, *mean*<br>• No significant differences in fasting glucose levels, *mean*	 C: 129.9; I: 129.7<br>C= 75.6; I: 77.3<br><br><br>C: 49.2; I: 19.8<br>C: 101.0; I: 108.4<br>C: 138.3; I: 139.2<br>C: 116.4; I: 126.3	 0.617<br>0.287<br><br><br>0.826<br>0.116<br>0.336<br>0.554
Nagykaldi Z, 2012[31]	• Increased proportion of patients receiving all recommended preventive services, *%*<br>o Increased proportion of patients taking low-dose aspirin, if indicated, *%*<br>o Increased proportion receiving Pneumococcal vaccine due to health conditions, *%*<br>o Increased proportion receiving Pneumococcal vaccine indicated due to age, *%*<br>o Increased proportion of children receiving all recommended immunisations, *%*	C: 67.6; I: 84.4<br>C: 52.3; I: 78.6<br>C: 53.9; I: 82.5<br>C: 44.6; I: 86.3<br>C: 87.2; I: 95.5	**p<0.0001**<br>**p<0.0001**<br>**p<0.0001**<br>**p<0.0001**<br>**p=0.044**
Krist AH, 2012[32]	Primary<br>• Higher increase in the proportion of patients up-to-date with all preventive services (16 M), *mean difference between groups*<br><br>Secondary:<br>• Subgroup analyses of users vs nonusers demonstrated significant differences in the increase of preventive services (%), *mean difference between groups*<br>o colorectal cancer screening (16M)<br>o breast cancer screening (4M)<br>o cervical cancer screening (16M)<br>o tetanus immunization (16M)<br><br>• No significant results for pneumococcal immunization, *mean difference between groups*	 +2.3<br><br><br><br>+14.3<br>+12.3<br>+13.4<br>+10.3<br><br>+8.9	 **p<0.05**<br><br><br><br>**p<0.02**<br>**p<0.05**<br>**p<0.02**<br>**p<0.02**<br><br>p>0.05

SAFETY

Ross SE, 2004[22]	Secondary:<br>• Increased general adherence, *mean difference between groups (95% CI)*<br>• No change in medication adherence, *mean difference between groups (95% CI)*	 +6.4 [1.8; 10.9]<br>+0.2 [-0.1; 0.6]	 **p=0.01**<br>p=0.15
Chrischilles EA, 2013[29]	Primary<br>• Higher likelihood of reporting differences between doctor and patient medication records, *n(%)*<br>• Higher likelihood of reporting a side effect, *n(%)*<br>• Higher likelihood of recognising a side effect, *n(%)*<br>• Higher likelihood to change list of current medications, *n(%)*<br>• Higher likelihood of asking the reason for medications on the list, *n(%)*<br>• Lower number of over the counter (OTC) drugs, *mean (SD)*<br>• Higher likelihood of starting a new prescription drug, mean *n(%)*<br>• Higher likelihood of stopping a prescription drug, *n(%)*<br>• Higher likelihood of changing the strength or dose of an OTC drug, *n(%)*<br>• No differences on self-reported adherence to medication, *n(%)*<br>• No differences on the number of prescription drugs, *mean (SD)*	 C: 53(8.4); I: 24(19.0)<br>C: 71(10.9);I: 29(22.8)<br>C: 462(72.1); 104(81.9)<br>C: 446(67.2); I: 113(88.3)<br>C: 135(34.6); I: 57(50.4)<br>C: 3.4(2.4); I: 4.3(2.9)<br>C: 145(21.5); I: 45(34.9)<br>C: 88(13.1); I: 35(27.1)<br>C: 12(1.8); I: 7 (5.4)<br>C: 13.8(2.0);I: 14.0(1.6)<br>C: 3.9(3.1); I: 4.6(3.1)	 **p<0.01**<br>**p<0.01**<br>**p<0.05**<br>**p<0.01**<br>**p<0.001**<br>**p<0.01**<br>**p<0.01**<br>**p<0.001**<br>**p<0.05**<br>**p<0.05**<br>p>0.05

	• No differences on the strength/dose of prescription drug, *n(%)*<br>• No differences on the likelihood of starting and OTC drug, *n(%)*<br>• No differences on the likelihood of stopping an OTC drug, n(%)<br>• No differences on the use of potentially inappropriate medications, n(%)<br>• No differences on taking 2 or more NSAID, n(%)<br>• No differences on the number of medication management problems, mean (SD)	C:89(13.2); I:21(16.3)<br>C: 54(8.0): I: 17(13.2)<br>C: 27(4.0);I: 10(7.8)<br>C: 129(19.2); I: 35(27.1)<br>C: 88(13.1);I: 25(19.4)<br>C: 1.4(1.4);I: 1.8(1.5)	p>0.05<br>p>0.05<br>p>0.05<br>p>0.05<br>p>0.05<br>p>0.05	
Schnipper JL, 2012[30]	Primary<br>• Decreased proportion of patients with discordant medications, *mean (SD)*<br>• Decreased number of discrepancies per patient with potential for severe harm, *mean (SD)*<br>• No changes on the number of discrepancies per patient with potential for harm, *mean (SD)*<br>• No changes in the number of preventable or ameliorable adverse drug events (ADEs) per patient, *mean (SD)*<br>• No changes on duration of ameliorable ADEs in days, *median (IQR)*	C: 451(30); I: 2(29)<br>C: 0.08(0.35); I: 0.03(0.22)<br>C: 0.34(0.85); I: 0.24 (0.62)<br>C: 0.14(0.47); I: 0.16(0.44)<br>C: 6(1-8); I: 5 (1-7)	**p=0.01**<br>**p=0.04**<br>p=0.059<br>p=0.45<br>p=0.35	◆
Quinn CC, 2008[19]	Secondary<br>• Increased proportion of patients having medication changed or titrated by their health care provider	C: 23; I: 84	**p=0.002**	◆

EFFICIENCY

Khan S, 2010[16]	Primary<br>• Fewer hospital inpatient admissions per subject, *mean*<br>• Shorted length of stay (days), *mean*<br>• Lower inpatient charges ($), *mean*<br>• Fewer emergency department visits, *mean*<br>• Lower emergency room charges, *mean*	C: 0.20; I: 0.17<br>C: 1.1; I:0.99<br>C: 3,480; I: 3,113<br>C: 0.36; I: 0.27<br>C: 414,30: I: 303.51	**p=0.01**<br>**p=0.01**<br>**p=0.02**<br>**p<0.0001**<br>**p<0.0001**	◆
Ross SE, 2004[22]	Secondary<br>• Higher number of emergency department visits, *n*<br>• No significant changes on the number of heart failure practice visits, *n*<br>• No significant changes on the number of hospitalisations, *n*	C: 8; I: 20<br>C: 325; I: 324<br>C: 21; I: 22	**p=0.03**<br>p=0.66<br>p=1.00	◆
Nagykaldi Z, 2012[31]	Secondary<br>• Fewer primary care visits, *mean*	C: 4.3; I: 2.9	**p<0.0001**	◆
Wagner PJ, 2012[24]	Secondary<br>• No differences on the number of outpatient visits, *mean (SD)*<br>• No differences in the number of hospital days, *mean (SD)*<br>• No differences in the number of emergency room visits, *mean (SD)*	C: 4.13(5.569); I: 4.16(3.629)<br>C: 0.39(1.686); I: 0.56(1.593)<br>C: 0.34(0.831); I: 0.42(0.934)	p=0.958<br>p=0.280<br>p=0.312	◆
Ralston JD, 2009[18]	Secondary<br>• No differences in the number of outpatient visits<br>• No differences in the number of primary care visits<br>• No difference in the number of specialist physician visits<br>• No differences on the number of inpatient days	C: -2.1 (7.0); I: 0.6(10.7)<br>C: -0.2(2.8): I: 0.0(2.9)<br>C: -1.9 (5.9); I: 0.6(9.0)<br>C: -0.3(1.8); I: 0.2(2.6)	p=0.18<br>p=0.76<br>p=0.14<br>p=0.32	◆
