# Supplementary figures and images for: Impact of providing patients access to electronic health records on quality and safety of care: a systematic review and meta-analysis
Source: BMJ Qual Saf. 2020 Jun 12;29(12):1019–32. doi: 10.1136/bmjqs-2019-010581 (PMC7785164; doi:10.1136/bmjqs-2019-010581)

HbA1c

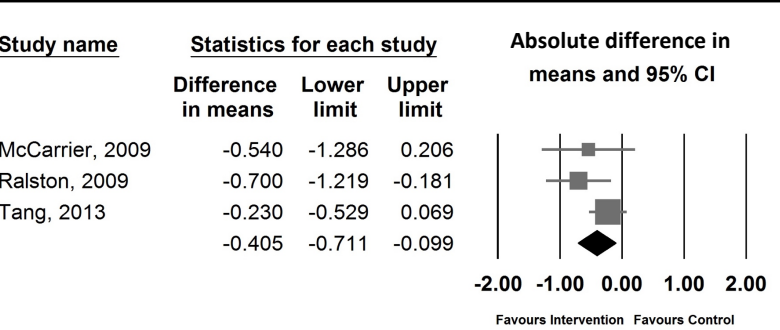

SBP

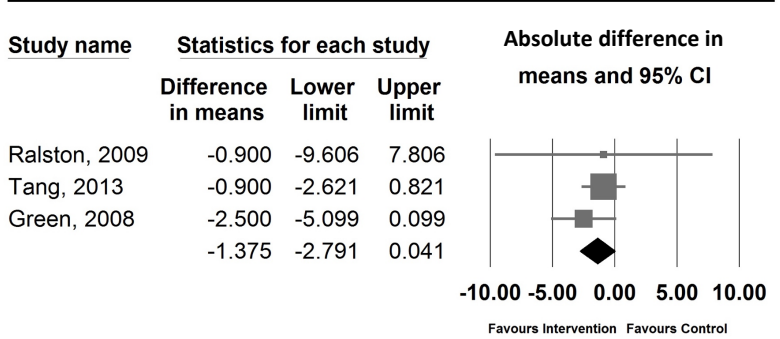

DBP

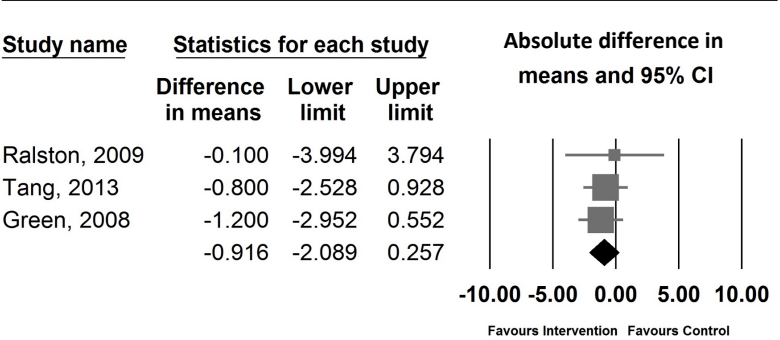

Supplement: Supplementary data [file bmjqs-2019-010581supp004.pdf]

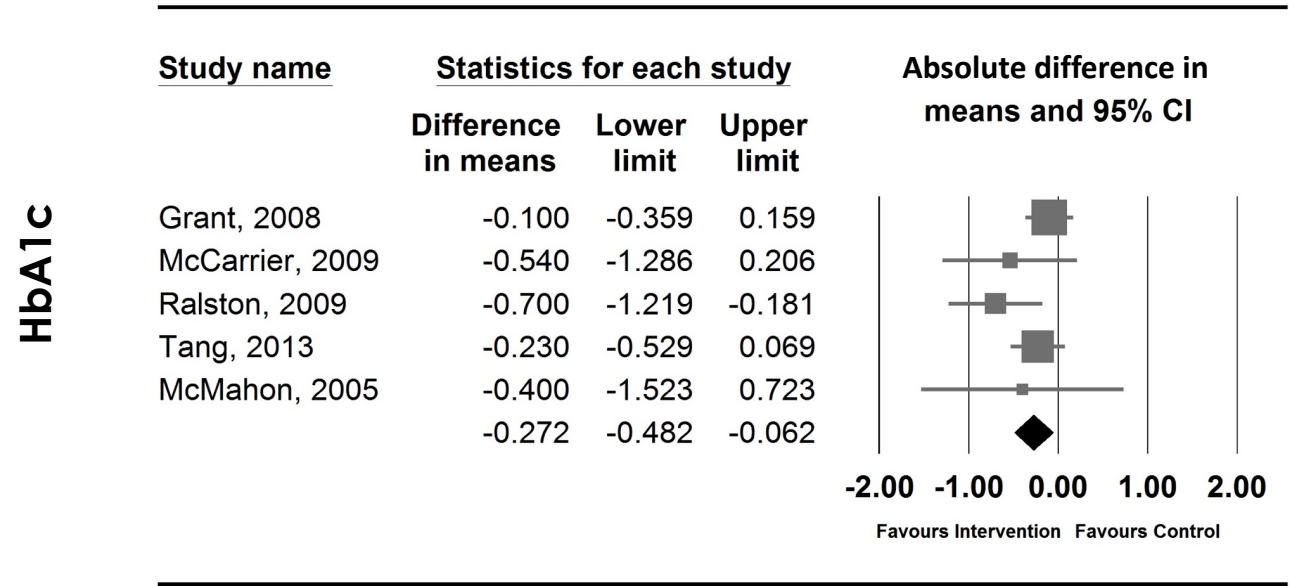

Supplement: Supplementary data [file bmjqs-2019-010581supp005.pdf]

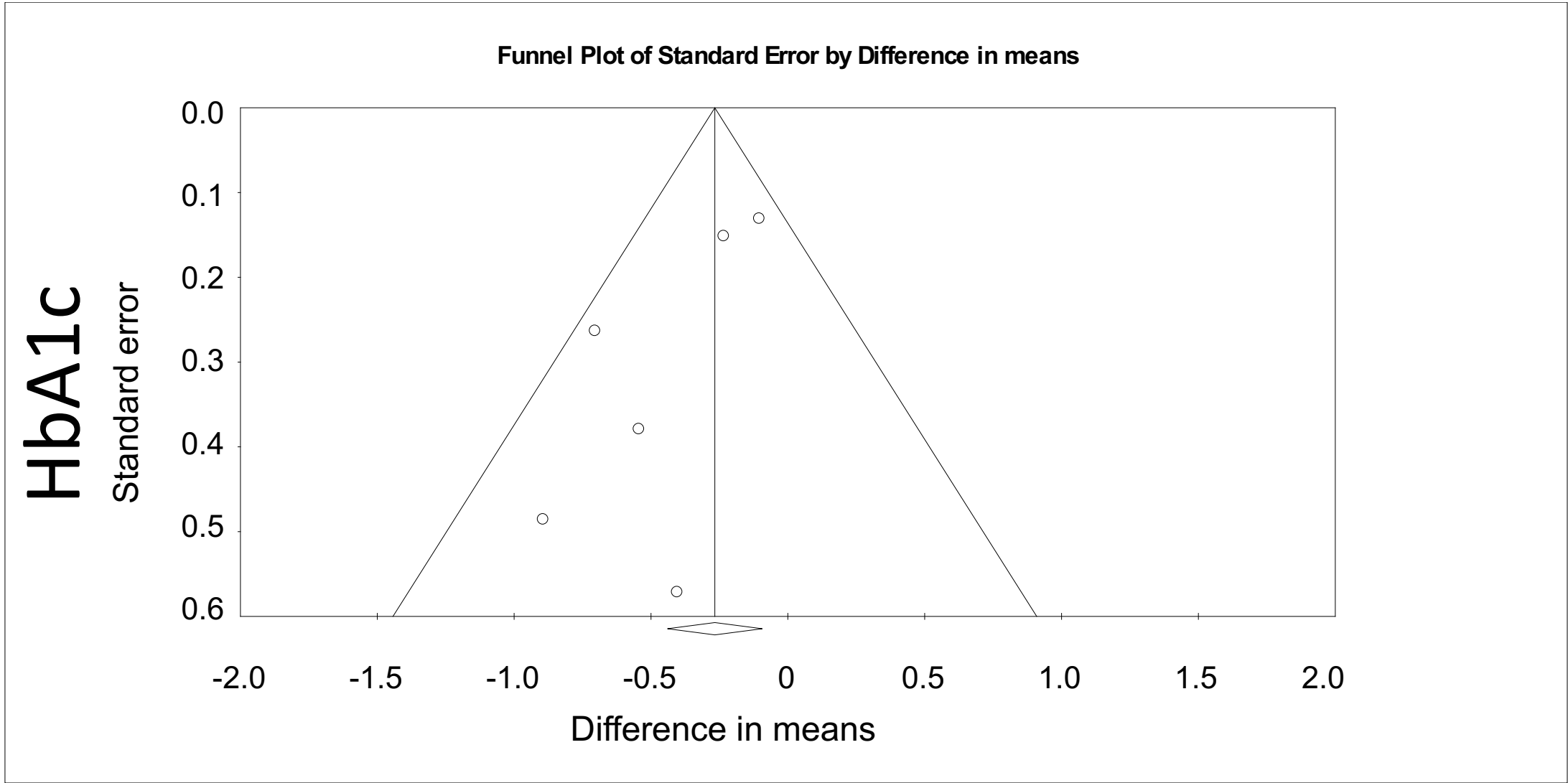

Supplement: Supplementary data [file bmjqs-2019-010581supp006.pdf]

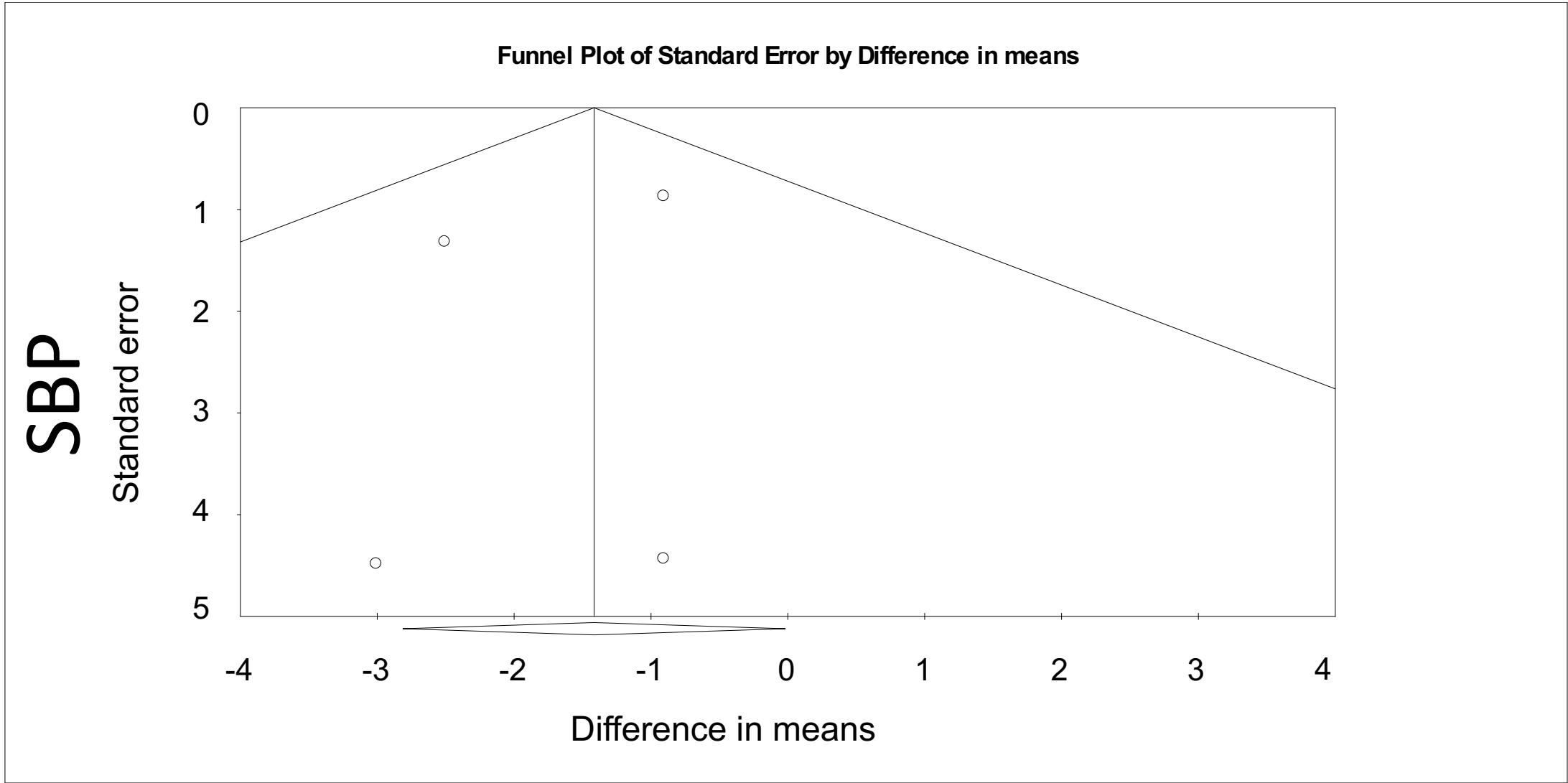

Supplement: Supplementary data [file bmjqs-2019-010581supp007.pdf]

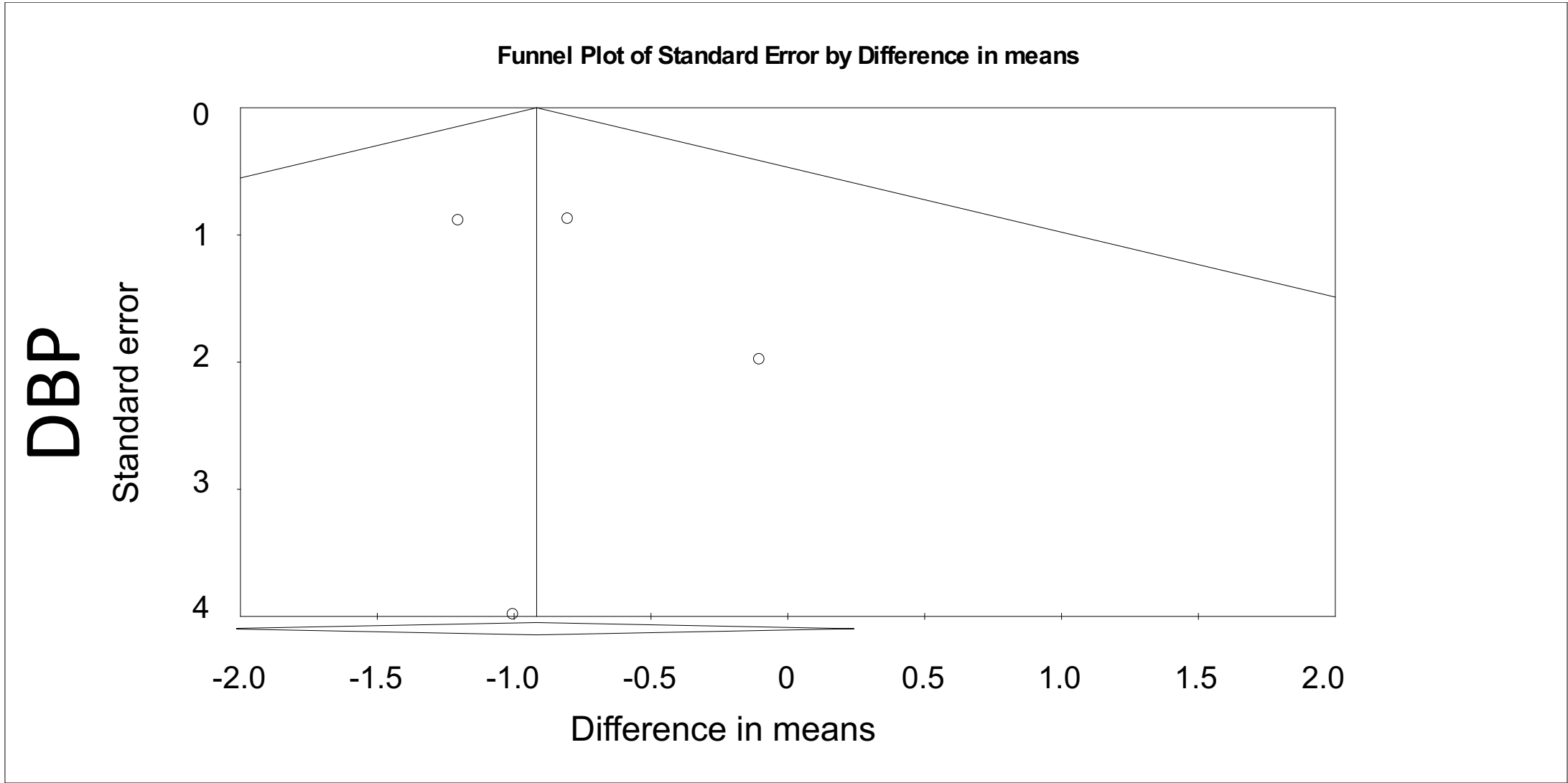

Supplement: Supplementary data [file bmjqs-2019-010581supp008.pdf]
